# Fertility preservation for girls and young women with cancer: population-based validation of criteria for ovarian tissue cryopreservation

**DOI:** 10.1016/S1470-2045(14)70334-1

**Published:** 2014-09

**Authors:** W Hamish B Wallace, Alice Grove Smith, Thomas W Kelsey, Angela E Edgar, Richard A Anderson

**Affiliations:** a Department of Haematology/Oncology, Royal Hospital for Sick Children, Edinburgh, UK; b Department of Child Life and Health, University of Edinburgh, Edinburgh, UK; c MRC Centre for Reproductive Health, University of Edinburgh, Edinburgh, UK; d School of Computer Science, University of St Andrews, St Andrews, UK

## Abstract

**Background:**

Ovarian tissue cryopreservation with later reimplantation has been shown to preserve fertility in adult women, but this approach remains unproven and experimental in children and adolescents. We aimed to assess the use of the Edinburgh selection criteria for ovarian tissue cryopreservation in girls and young women with cancer to determine whether we are offering this invasive procedure to the patients who are most at risk of premature ovarian insufficiency.

**Methods:**

Cryopreservation of ovarian tissue has been selectively offered to girls and young women with cancer who met the Edinburgh selection criteria since 1996. Between Jan 1, 1996, and June 30, 2012, 410 female patients younger than 18 years at diagnosis were treated for cancer (including leukaemia and brain tumours) at the Edinburgh Children's Cancer Centre, which serves the whole South East of Scotland region. We determined the ovarian status of these patients from review of clinical records and classified them as having premature ovarian insufficiency or not, or as unable to be determined. Patients younger than 12 years at time of data cutoff (Jan 31, 2013) were excluded from the analysis.

**Findings:**

34 (8%) of the 410 patients met the Edinburgh selection criteria and were offered ovarian tissue cryopreservation before starting cancer treatment. 13 patients declined the procedure and 21 consented, and the procedure was completed successfully in 20 patients. Of the 20 patients who had ovarian tissue successfully cryopreserved, 14 were available for assessment of ovarian function. Of the 13 patients who had declined the procedure, six were available for assessment of ovarian function. Median age at the time of follow-up for the 20 assessable patients was 16·9 years (IQR 15·5–21·8). Of the 14 assessable patients who had successfully undergone ovarian cryopreservation, six had developed premature ovarian insufficiency at a median age of 13·4 years (IQR 12·5–14·6), one of whom also had a natural pregnancy. Of the six assessable patients who had declined the procedure, one had developed premature ovarian insufficiency. Assessment of ovarian function was possible for 141 of the 376 patients who were not offered cryopreservation; one of these patients had developed premature ovarian insufficiency. The cumulative probability of developing premature ovarian insufficiency after treatment was completed was significantly higher for patients who met the criteria for ovarian tissue cryopreservation than for those who did not (15-year probability 35% [95% CI 10–53] *vs* 1% [0–2]; p<0·0001; hazard ratio 56·8 [95% CI 6·2–521·6] at 10 years).

**Interpretation:**

The results of this analysis show that the Edinburgh selection criteria accurately identify the few girls and young women who will develop premature ovarian insufficiency, and validate their use for selection of patients for ovarian tissue cryopreservation. Further follow-up of this cohort of patients is likely to allow refinement of the criteria for this experimental procedure in girls and young women with cancer.

**Funding:**

UK Medical Research Council.

## Introduction

Childhood cancer survival has improved greatly over the past five decades. About 80% of children with cancer will be alive 5 years after diagnosis and about 70% will be alive and cured of their original cancer 10 years after diagnosis.^1^ However, some cancer treatments can compromise ovarian function in children.^2^, ^3^, ^4^ The increasing life expectancy of children with cancer has led to a growing population of girls and young women who are at risk of developing premature ovarian insufficiency. Most girls and young women treated for cancer will retain a window of opportunity for fertility; however, for those at high risk of premature ovarian insufficiency the options for fertility preservation are few and experimental.

Ovarian tissue cryopreservation is an important development for fertility preservation in girls and young women at risk of premature ovarian insufficiency as a result of treatment for cancer.^5^, ^6^, ^7^, ^8^ At least 30 pregnancies after orthotopic reimplantation of frozen–thawed ovarian cortex have been reported worldwide,^5^ showing that this approach is viable in adults, although the success rate is unclear because the total number of women in whom frozen–thawed ovarian tissue has been reimplanted is unknown. The procedure remains unproven and experimental in girls and young women.

Evidence from case series and reviews^9^, ^10^ has suggested that the collection of ovarian tissue for freezing by laparoscopy under a general anaesthetic is safe and feasible in prepubertal girls as well as in adult women. The procedure is invasive, and can carry an unacceptable operative risk in some children with cancer who might be immunocompromised and pancytopenic and are therefore at increased risk of bleeding and infection. Haematological cancer in particular can involve the ovary,^11^, ^12^ precluding later replacement of tissue to restore fertility. These uncertainties, and the unknown effectiveness of the procedure for the restoration of fertility, dictate that it should only be offered to girls and young women who are at high risk of premature ovarian insufficiency and who will have a substantially reduced opportunity for fertility. Accurate identification of these patients requires that the risk of premature ovarian insufficiency, which is determined by the nature of the treatment to be delivered and not the disease itself, can be assessed at the time of diagnosis.^4^ However, in children, the full consequences of treatment with respect to reproductive function are not fully known and will take several decades to be fully realised.^13^, ^14^ Thus an accurate fertility prognosis cannot always be obtained before the start of treatment. On the basis of these considerations, we have previously proposed that factors that affect the assessment of an individual patient for invasive fertility preservation techniques can be usefully grouped as intrinsic (ie, related to the patient herself and her present state of health) and extrinsic (ie, related largely to the anticipated treatment and the availability of appropriate expertise for the techniques proposed).^15^

With the now proven success of ovarian tissue cryopreservation in adult women,^5^ it is important to establish if this experimental technique is being offered to the correct young patients—ie, those who are at high risk for the development of premature ovarian insufficiency, can safely undergo laparoscopic surgery, and have a good long-term prognosis. The Edinburgh selection criteria for ovarian tissue cryopreservation have been previously reported,^4^, ^9^ having been developed after multidisciplinary discussion in 1996 and slightly revised in 2000. The aim of this study is to assess the accuracy of patient selection for ovarian tissue cryopreservation in a single centre that has offered this experimental procedure (as part of this approved study) to selected children and teenagers with cancer since 1996, by comparing the prevalence of premature ovarian insufficiency in those who were and were not offered the procedure.

## Methods

### Study design and participants

The study cohort consisted of all female patients treated for cancer (including leukaemia and brain tumours) at the Edinburgh Children's Cancer Centre who were younger than 18 years at diagnosis between Jan 1, 1996, and June 30, 2012. We aimed to assess the relation between whether or not patients had been offered ovarian tissue cryopreservation and whether they had premature ovarian insufficiency at the time of their most recent assessment. A risk assessment for fertility preservation was made for all patients before the start of treatment,^15^ and cryopreservation of ovarian tissue was offered to those patients who met the Edinburgh selection criteria (panel 1). These criteria were developed after discussion within the multidisciplinary team and were approved by a research ethics committee. We used knowledge of the relevant scientific literature and our own experience to make an initial assessment at diagnosis of whether an individual patient with a new diagnosis of cancer had a greater than 50% risk of premature ovarian insufficiency. The development of a treatment plan for each patient allowed a decision about the risk of premature ovarian insufficiency. Most of the patients identified as being at high risk of premature ovarian insufficiency were planned to be treated with high-dose alkylating agent-based regimens or radiotherapy to a field that would include the ovaries. We obtained written informed consent from parents and, where possible, patients for ovarian tissue cryopreservation.

Panel 1 **The Edinburgh selection criteria**

• Age younger than 35 years
• No previous chemotherapy or radiotherapy if aged 15 years or older at diagnosis, but mild, non-gonadotoxic chemotherapy acceptable if younger than 15 years
• A realistic chance of surviving for 5 years
• A high risk of premature ovarian insufficiency (>50%)
• Informed consent (from parents and, where possible, patient)
• Negative serology results for HIV, syphilis, and hepatitis B
• Not pregnant and no existing children

This study was done with the approval of the Lothian Research Ethics Committee.

### Procedures

In those patients who consented to the procedure, ovarian tissue was obtained laparoscopically under general anaesthetic and cryopreserved. Generally, three to five ovarian cortical strips from one ovary were dissected with scissors, without diathermy, with the aim of removing roughly 70% of the ovarian cortex. Thin strips (not more than 1·5 mm thick) were collected into Liebovitz medium and cryopreserved as previously described.^16^ A whole ovary could be obtained from very young patients for whom the cortex could not be dissected. A further small biopsy sample was taken in all cases for pathological examination to investigate the possibility of malignant contamination. Biopsy samples are stored in the vapour phase of liquid nitrogen at the Scottish National Blood Transfusion Service Tissue Establishment (Edinburgh, UK) under licence from the UK Human Tissue Authority and, for part of the time period reported, the Human Fertilisation and Embryology Authority.

Ovarian status was recorded for the whole surviving cohort who were aged 12 years or older on Jan 31, 2013, from review of their medical notes from diagnosis and when they attended routine follow-up assessments. A typical patient is seen 24 times in the 5 years after completion of treatment; thereafter all patients are seen at least annually. Premature ovarian insufficiency was defined as the documented presence of at least two of three identifying features: amenorrhoea for at least 4 months; serum follicle stimulating hormone greater than 25 IU/L on at least two occasions; and low (<150 pmol/L) serum oestradiol concomitant with raised (>25 IU/L) follicle stimulating hormone. The absence of premature ovarian insufficiency was confirmed by one or more of three identifying features: premenarcheal, but progressing normally through puberty; having regular menses while not taking hormonal contraception; or normal concentrations of gonadotropins and oestradiol. Ovarian status could not be determined in children younger than 12 years, because the measurement of gonadotropins and oestradiol in girls younger than 12 years is not a reliable measure of ovarian function, or in individuals taking hormonal contraception.

### Statistical analysis

We used the Kaplan-Meier method^17^ with premature ovarian insufficiency as the defined event. Since patient classification into the premature ovarian insufficiency group occurred after the actual date of onset of premature ovarian insufficiency, we discretised the 15 years after diagnosis into calendar years, and recorded each assessment of premature ovarian insufficiency occurring in the year after diagnosis as a premature ovarian insufficiency event in that year. We then calculated life tables for the patients who were offered ovarian tissue cryopreservation (the offered group) and those who were not offered the procedure (the not-offered group) as cumulative event-free probabilities for each year of follow-up after diagnosis.

We derived p values to test the null hypothesis that the frequency of premature ovarian insufficiency occurrences were distributed evenly between the two groups. We also calculated hazard ratios (HRs) to quantify any differences in the proportion of patients with premature ovarian insufficiency between the two groups. The HR for each year was calculated as the ratio of observed-to-expected occurrences of premature ovarian insufficiency in the offered group in that year, divided by the ratio of observed-to-expected occurrences of premature ovarian insufficiency in the not-offered group in that year, with the observed and expected values taken from the intermediate calculations used to derive the Kaplan-Meier cumulative probabilities.

We calculated HRs using Microsoft Excel for Mac (version 14.3.7) and did the Kaplan-Meier analyses, including calculation of CIs and p values, using the KMsurv version 0.1-5 package for R version 2.15.3.

### Role of the funding source

The funder of the study had no role in study design, data collection, data analysis, data interpretation, or writing of the report. All authors had access to the raw data (apart from TWK, who had access to anonymised raw data). The corresponding author had full access to all the data in the study and had final responsibility for the decision to submit for publication.

## Results

**Figure 1:**
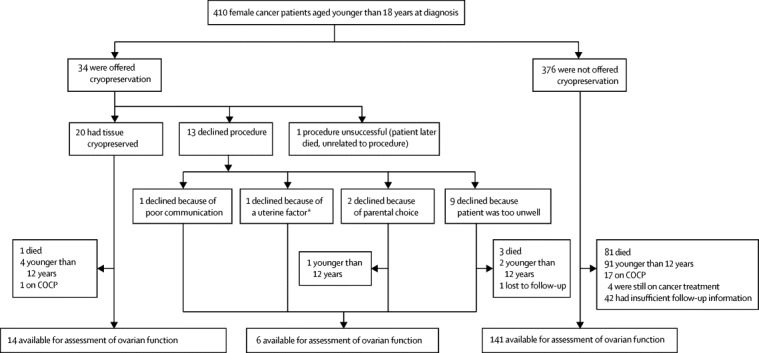
Study profile COCP=combined oral contraceptive pill. *Treatment plan meant that there was a high chance that the patient would receive radiation to her pelvis and uterus at a very young age.

The eligible study population consisted of 410 patients (figure 1). Ovarian status could not be determined in children younger than 12 years (n=98), and 18 individuals taking hormonal contraception (combined oral contraceptive pill in all cases) were excluded from analyses. At the time of data censoring (Jan 31, 2013), an additional four patients who had not been offered ovarian tissue cryopreservation were still receiving cancer treatment, and we were unable to classify ovarian function in a further 42 patients in the same group because of incomplete information in their medical records. Median follow-up for the assessable patients who were offered and underwent ovarian tissue cryopreservation (n=14) was 6·0 years (IQR 3·5–14·9); for those who were offered the procedure but declined (n=6) it was 10·9 years (7·4–13·7) and for those who were not offered the procedure (n=141) it was 10·7 years (6·1–13·8).

Ovarian tissue cryopreservation was offered to 34 patients, of whom 21 underwent the procedure and 13 declined. 376 patients were not offered the procedure. The median age of the cohort of patients who were offered cryopreservation and were assessable (n=20, figure 1) was 16·9 years (IQR 15·5–21·8) at data cutoff for this analysis; the median age of assessable patients in the not-offered group (n=141) was 17·9 years (15·6–22·0). The procedure was unsuccessful in one individual (the second case undertaken) because of technical problems with surgical equipment, with no adverse effect on the patient. Of the patients who underwent ovarian cryopreservation, a whole ovary was obtained in the two youngest patients (table); the other patients had ovarian cortical strips taken from one ovary.

**Table:** Characteristics and ovarian function for patients who underwent laparoscopy for fertility preservation, by patient number

	**Diagnosis**	**Age at cryopreservation (years)**	**Method of ovarian tissue collection**	**Complications from procedure**	**Duration since cryopreservation (years)**	**Age at last assessment (years)**	**Ovarian function**
1	Hodgkin's lymphoma*	14·9	Laparoscopic cortical strip	None	15·8	30·2	Not POI
2	Ewing's sarcoma	9·2	Procedure failed	None	..	..	Deceased
3	Ewing's sarcoma (pubic bone)	14·9	Laparoscopic cortical strip	None	16·6	25·6	POI (plus one child)
4	Sacral ependymoma	11·3	Laparoscopic cortical strip	None	15·8	24·5	Not POI
5	Hodgkin's lymphoma	13·7	Laparoscopic cortical strip	None	15·6	28·9	Not POI
6	Hodgkin's lymphoma	11·0	Laparoscopic cortical strip	None	14·7	..	On COCP
7	Chronic granulocytic leukaemia	9·9	Laparoscopic cortical strip	None	12·2	21·7	Not POI
8	Rhabdomyosarcoma	5·3	Laparoscopic cortical strip	None	8·2	13·1	POI
9	Ewing's sarcoma (pelvic)	9·8	Laparoscopic cortical strip	None	6·7	15·6	POI
10	Uterine cervix rhabdomyosarcoma†	16·4	Laparoscopic cortical strip	None	5·1	17·5	Not POI
11	Hodgkin's lymphoma‡	14·0	Laparoscopic cortical strip	None	3·2	17·2	POI
12	Abdominal embryonal rhabdomyosarcoma	7·9	Laparoscopic cortical strip	None	..	..	Deceased
13	Ewing's sarcoma	12·1	Laparoscopic cortical strip§	None	3·9	15·2	POI
14	Hodgkin's lymphoma	12·7	Laparoscopic cortical strip	None	3·3	14·3	POI
15	Metastatic medulloblastoma	8·1	Laparoscopic cortical strip	None	2·9	..	Not assessed
16	Hodgkin's lymphoma	15·2	Laparoscopic cortical strip	None	1·9	16·9	Not POI
17	Alveolar rhabdomyosarcoma	10·5	Laparoscopic cortical strip	None	1·4	..	Not assessed
18	Embryonal rhabdomyosarcoma	3·0	Oophorectomy	None	1·4	..	Not assessed
19	Ewing's sarcoma	12·0	Laparoscopic cortical strip	None	1·4	13·5	Not POI
20	Undifferentiated sarcoma	12·3	Laparoscopic cortical strip§	None	1·0	13·4	Not POI
21	Wilms' tumour	1·2	Oophorectomy	None	0·6	..	Not assessed

All tissue was collected before chemotherapy (with or without radiotherapy) were given, apart from patients 1 and 11. Ovarian function was not assessed in patients who were younger than 12 years at the time of data censoring. POI=premature ovarian insufficiency. COCP=combined oral contraceptive pill.

* Tissue collected after relapse of disease 21 months after initial radiotherapy.

† Diagnosis changed to Müllerian adenosarcoma shortly after tissue cryopreservation.

‡ Tissue collected after relapse of disease 7 months after initial radiotherapy.

§ Metastatic deposits identified on cortical strip.

Of the 20 patients in the offered group who had ovarian tissue successfully cryopreserved, four were younger than 12 years at the time of last follow-up, one had died, and one was using hormonal contraception; 14 patients were aged 12 years or older and available for assessment of ovarian function. Of these 14, six had developed premature ovarian insufficiency at a median age of 13·4 years (IQR 12·5–14·6), after a median interval since diagnosis of 1·7 years (0·8–2·4), and eight had normal ovarian function at a median age of 21·9 years (16·2–27·7). One patient who developed premature ovarian insufficiency subsequently had a successful natural pregnancy, as reported previously.^18^

Of the 13 patients who were offered cryopreservation but declined the procedure (figure 1), three were deceased and three were younger than 12 years at the time of last follow-up; one was lost to follow-up. Therefore, six were available for assessment, of whom one had developed premature ovarian insufficiency at age 13·4 years (interval 7·9 years). Median age of the five patients without premature ovarian insufficiency was 16·7 years (IQR 15·4–17·9). Thus premature ovarian insufficiency was identified in a total of seven (35%) of 20 assessable patients in the group offered ovarian cryopreservation.

Of the 376 patients not offered ovarian cryopreservation, 81 were deceased at the time of assessment, 91 were younger than 12 years, 17 were using hormonal contraception, four were still receiving cancer treatment, and information about ovarian status was incomplete for 42 (figure 1). Of the 141 who were not offered cryopreservation and for whom ovarian function is known, 140 did not have premature ovarian insufficiency at a median age 17·9 years (IQR 15·6–22·0). Of these, 112 had achieved menarche, 17 were premenarchal but aged 12 years or older and were progressing normally through puberty, and 11 had hypogonadotropic hypogonadism as a result of cranial irradiation treatment for brain tumours. Premature ovarian insufficiency was confirmed in only one patient in this group, who developed premature ovarian insufficiency at age 15·0 years (2·9 years after diagnosis) after a successful allogeneic bone marrow transplantation for relapsed acute myeloid leukaemia with busulfan and cyclophosphamide conditioning. She was not offered cryopreservation of ovarian tissue at the time of original diagnosis because she did not meet the criteria (low risk of premature ovarian insufficiency), nor at relapse because of the urgency of the need for further treatment, when she was believed to be too unwell to undergo a laparoscopic procedure.

**Figure 2:**
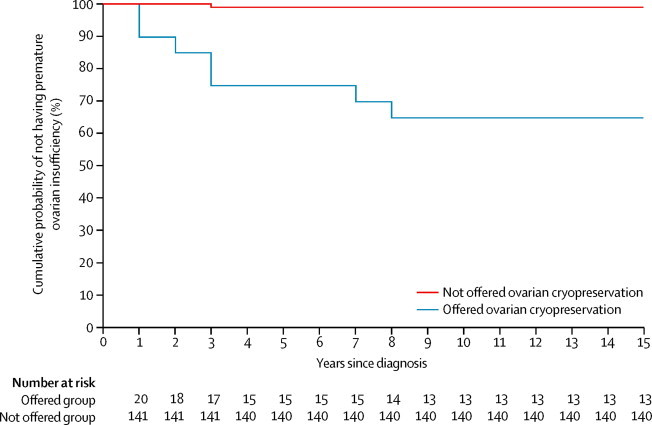
Cumulative probabilities of not having premature ovarian insufficiency

Therefore, by 9 years from diagnosis (the interval to the last premature ovarian insufficiency event), seven (35%) of 20 patients offered ovarian tissue cryopreservation had developed premature ovarian insufficiency, compared with one (1%) of 141 of those not offered ovarian tissue cryopreservation. The cumulative probability of developing premature ovarian insufficiency after treatment was completed was significantly higher for the patients offered ovarian tissue cryopreservation than for those who were not offered ovarian tissue cryopreservation (15-year probability 35% [95% CI 10–53] *vs* 1% [0–2]; p<0·0001; HR 56·8 [95% CI 6·2–521·6] at 10 years; figure 2).

In addition to the assessed risk of premature ovarian insufficiency as a result of the planned treatment, another of the Edinburgh selection criteria is that the patient has to have a realistic chance of surviving for 5 years (panel 1). In our cohort, four (12%) of 34 patients in the offered group (or one [5%] of 20 who had their ovarian tissue cryopreserved) were deceased at the time of analysis, compared with 81 (22%) of 376 in the not-offered group; thus, overall this criterion was met.

13 patients declined the offer of ovarian tissue cryopreservation. The most common reason for this decision (in nine cases) was that both the medical team and the patient's parents believed that she was too unwell to undergo an additional laparoscopic procedure. In two patients, the parents declined to proceed with ovarian tissue cryopreservation for no specific reason when it was believed to be medically appropriate. For another patient, severe language difficulties contributed to the investigators being unable to obtain informed consent for the procedure. For the remaining patient, who had a vaginal rhabdomyosarcoma, the parents decided against the procedure because there was a high chance that their daughter would receive radiation to her pelvis and uterus at a very young age, making it highly unlikely that she would be able to carry a pregnancy successfully in the future.

## Discussion

The results of our analysis show that the Edinburgh selection criteria accurately predict which girls and young women will or will not develop premature ovarian insufficiency. These findings validate the use of these criteria for patient selection for ovarian tissue cryopreservation before the start of cancer treatment.

High survival following childhood cancer has led to an increased focus on the late effects of treatment,^19^, ^20^ and the effect of cancer treatment on fertility is a prime concern to young women.^21^ Although several options might be available to adult women, including ovarian tissue, oocyte, and embryo cryostorage, none are established in girls and adolescents, although ovarian tissue cryopreservation has been proposed.^9^, ^10^ Because patient selection is crucial for this experimental procedure and the outcome might not be known for many years, we have analysed 15 years of experience in a regional children's cancer centre. Applying the Edinburgh selection criteria, ovarian tissue cryopreservation was offered to 8% of our patients since 1996, and done in 5%. The procedure was safe and without surgical complications. Through the application of our criteria, we have offered ovarian tissue cryopreservation to all but one of our patients who have gone on develop premature ovarian insufficiency so far. We believe that the Edinburgh selection criteria provide a solid scientific basis for the identification of patients at risk of premature ovarian insufficiency and to whom this invasive procedure may be offered (panel 2).

Panel 2 **Research in context**

**Systematic review**

For prepubertal girls and young women with cancer who are unwilling to delay the start of chemotherapy, cryopreservation of ovarian tissue is the only fertility preservation option available. So far, 30 livebirths have been reported after reimplantation of cryopreserved ovarian tissue in adult women:^5^ none have been reported using ovarian tissue from prepubertal girls. Most girls and young women treated for cancer will retain a window of opportunity for fertility in the future once they have survived their original cancer, dependent on the nature of the patients' planned treatment (which might change dependent on their response).^4^ The identification of those patients who are at the highest risk of loss of fertility, thus justifying the use of an invasive experimental approach, is crucial. No previous study has addressed the accuracy and safety with which this procedure can be offered to girls and adolescents with cancer who are at highest risk of developing premature ovarian insufficiency.

**Interpretation**

We have shown that the Edinburgh selection criteria predict which young female patients with cancer are more likely to develop premature ovarian insufficiency and are therefore most likely to benefit from ovarian tissue cryopreservation. The procedure is invasive, requiring surgery, and the success rate in terms of future livebirths remains unknown. A minority of girls and young women with cancer are at high risk of premature ovarian insufficiency and, because this approach remains experimental,^7^ it is necessary to limit ovarian tissue cryopreservation to those patients at high risk of premature ovarian insufficiency. Future research should focus on the development of this new and experimental service for girls and young women with cancer who are at highest risk of premature ovarian insufficiency, but who have a realistic chance of survival.

Some girls and young women treated for cancer are at risk of premature ovarian insufficiency and infertility. Overall, data from the Childhood Cancer Survivor Study^14^ suggest that the relative risk of premature ovarian insufficiency is roughly 13 by age 40 years, compared with sibling controls, although 92% of survivors did not have premature ovarian insufficiency by that age.^22^ However, survivors who have not developed premature ovarian insufficiency are still at increased risk of infertility,^14^ as are women treated for cancer in adulthood.^23^ Accurate identification of girls and young women at high risk of premature ovarian insufficiency is important so as to allow the application of an experimental technique to those patients most likely to benefit in the long term.

We have offered ovarian tissue cryopreservation for more than 15 years to selected female patients at our regional children's cancer centre. The key selection criteria are the expected effect of proposed treatment on ovarian function, and the likelihood of survival, as well as surgical considerations with respect to the safety of the procedure. The effect of treatment can be classified by risk,^24^ with high-risk treatment involving high doses of alkylating agent-based treatment or abdominopelvic irradiation. Development of the treatment plan in all cases allowed a decision about the risk of premature ovarian insufficiency, but disease response or relapse will often require the treatment plan to be revised. This issue is exemplified by the single patient in the group not offered cryopreservation who developed premature ovarian insufficiency: she did not meet the criteria for ovarian tissue cryopreservation at the time of original diagnosis, but when she presented with a relapse she was believed to be too unwell to justify the surgical intervention needed. Similarly, the patient with cervical rhabdomyosarcoma shows the importance of the contemporaneous nature of the decision. She was offered ovarian tissue cryopreservation because of her initial pathological diagnosis, with the intended treatment including use of an alkylating agent and radiation to her pelvis. The ovarian tissue cryopreservation procedure was done successfully, but shortly after the procedure the diagnosis was changed to Müllerian adenosarcoma, and she did not need any potentially gonadotoxic treatment. This patient was therefore not at high risk of premature ovarian insufficiency, but she is nevertheless included in this analysis because the initial assessment was made on the basis of the initial (later revised) diagnosis; the key issue is that the assessment has to be based on the intended treatment plan.

Although the duration of follow-up in the present analysis is up to 16·6 years, for many of the patients the duration is much less (as little as a few months), so additional young women survivors might develop premature ovarian insufficiency with time. Continued follow-up is therefore necessary to test whether the substantial difference in prevalence of premature ovarian insufficiency between the group offered cryopreservation and that not offered it at this point persists, or perhaps widens further, and to allow assessment of other outcomes such as fertility.

Although the diagnosis of premature ovarian insufficiency used in our analysis is robust, the data are limited by the large number of patients for whom ovarian function could not be determined (because some patients were still at a prepubertal age, some were using hormonal contraception, and some did not have information about ovarian function in their medical records). The diagnosis of premature ovarian insufficiency requires measurement of follicle stimulating hormone and oestradiol, which are unreliable in prepubertal girls. Anti-Müllerian hormone is not established as part of the diagnosis of premature ovarian insufficiency, but might become a useful biomarker in children because it is measurable in girls of all ages and shows a progressive rise through childhood.^25^ The concentration of anti-Müllerian hormone falls during cancer treatment in prepubertal and adolescent girls^13^ as it does in adults,^26^ with clearly divergent patterns of recovery after treatment completion in accordance with gonadotoxicity risk.^13^, ^27^ Measurement of anti-Müllerian hormone even in prepubertal cancer survivors might thus be of diagnostic value for the identification of individuals with premature ovarian insufficiency in whom early sex steroid treatment can be introduced for induction of puberty.

The inability to confirm or refute premature ovarian insufficiency in some women might have biased our analysis. The proportion of prepubertal girls was similar in the groups who were and were not offered cryopreservation. All women taking hormonal contraception were taking it to prevent pregnancy rather than as hormone replacement, although premature ovarian insufficiency might have developed after the start of oral contraceptive use in some individuals, and therefore gone undetected in our analysis. In patients with an incomplete record, evidence of premature ovarian insufficiency would probably have been noted, whereas normal ovarian function might not have been recorded. Thus, although some individuals within the group not offered cryopreservation (prepubertal, oral contraceptive users, or those with incomplete information about ovarian function) might have premature ovarian insufficiency that we were unable to identify, identification of these cases would be very unlikely to give a similar prevalence of premature ovarian insufficiency to that in the group who were offered the procedure.

Reimplantation of ovarian tissue has resulted in successful pregnancies in some adult women, confirming the potential of this approach.^5^ No cases have been reported of pregnancy after ovarian tissue cryopreservation in childhood or adolescence. However, findings from a recent study in an ovine model^28^ have shown that grafting immature ovarian tissue can restore spontaneous puberty and fertility,^28^ and a mature oocyte at metaphase II has been derived from a prepubertal ovarian biopsy sample xenografted into a mouse.^29^ In our study population, none of the young women has yet requested use of their stored tissue, nor are we aware of such use for fertility elsewhere. The authors of two case reports^30^, ^31^ have described the replacement of ovarian tissue in adolescents for the purpose of oestrogen production for pubertal induction;^30^, ^31^ we do not believe that the use of such a scarce resource is appropriate for this purpose.^32^ The evidence of successful (if short-lived) hormone production by the ovarian autografts in those reports does, however, suggest that follicular development is possible, potentially allowing for oocyte maturation, ovulation, and fertility.

In conclusion, our data show that specific selection criteria can be applied at diagnosis to girls and young women with cancer to identify the fairly small proportion of patients who are likely to survive but are at high risk of premature ovarian insufficiency. Whether ovarian tissue cryopreservation can give an opportunity for fertility in the future for these patients still needs to be determined.
